# Crystal structure of (μ-1,4-di­carb­oxy­butane-1,4-di­carboxyl­ato)bis­[bis­(tri­phenyl­phosphane)silver(I)] di­chloro­methane tris­olvate

**DOI:** 10.1107/S2056989016000797

**Published:** 2016-01-23

**Authors:** Peter Frenzel, Marcus Korb, Heinrich Lang

**Affiliations:** a Technische Universität Chemnitz, Fakultät für Naturwissenschaften, Institut für Chemie, Anorganische Chemie, D-09107 Chemnitz

**Keywords:** crystal structure, silver, tri­phenyl­phosphane, tetra­carb­oxy­lic acid, hydrogen bond, bridges

## Abstract

The mol­ecular structure of the tetra­kis(tri­phenyl­phosphan­yl)disilver salt of butane-1,1,4,4-tetra­carb­oxy­lic acid, [Ag_2_(C_8_H_8_O_8_)(C_18_H_15_P)_4_]·3CH_2_Cl_2_, crystallizes with one and a half mol­ecules of di­chloro­methane in the asymmetric unit. The coordination complex exhibits an inversion centre through the central CH_2_—CH_2_ bond. The Ag^I^ atom has a distorted trigonal–planar P_2_O coordination environment. The packing is characterized by inter­molecular *T*-shaped π–π inter­actions between the phenyl rings of the PPh_3_ substituents in neighbouring mol­ecules, forming a ladder-type superstructure parallel to [010]. These ladders are arranged in layers parallel to (101). Intra­molecular hydrogen bonds between the OH group and one O atom of the Ag-bonded carboxyl­ate group results in an asymmetric bidendate coordination of the carboxyl­ate moiety to the Ag^I^ ion.

## Abstract 2

The mol­ecular structure of the distorted trigonal–planar-coordinated tetra­kis­(tri­phenyl­phosphan­yl)disilver salt of butane-1,1,4,4-tetra­carb­oxy­lic acid is reported, present as a di­chloro­methane tris­olvate. The coordination complex exhibits an inversion centre through the CH_2_—CH_2_ bond and inter­molecular *T*-shaped π–π inter­actions between the phenyl rings of the PPh_3_ substituents, forming a ladder-type superstructure parallel to the *b* axis.

## Chemical context   

Silver precursors [*e.g*. silver(I) carboxyl­ates and silver(I) *β*-diketonates] exhibit a wide range of applications, for instance the formation of thin, metallic layers by means of CVD (Chemical Vapour Deposition) or CCVD (Combustion Chemical Vapour Deposition) (Struppert *et al.*, 2010 ▸; Jakob *et al.*, 2006 ▸; Schmidt *et al.*, 2005 ▸; Lang & Buschbeck, 2009 ▸; Lang, 2011 ▸; Lang & Dietrich, 2013 ▸; Chi & Lu, 2001 ▸), spin coating (Jakob *et al.*, 2010 ▸) or inkjet printing (Jahn *et al.*, 2010*a* ▸,**b* ▸;* Gäbler *et al.*, 2016 ▸). The respective silver layers show closed and homogeneous silver films and therefore possess a good conductivity. In addition, silver carboxyl­ates such as [AgO_2_C*R*]_*n*_ (*n* is the degree of aggregation) allow for the formation and stabilization of silver nanoparticles, which can, for example, be used for catalytic processes (Steffan *et al.*, 2009 ▸). They are also used in biological studies (Fourie *et al.*, 2012 ▸; Langner *et al.*, 2012 ▸).
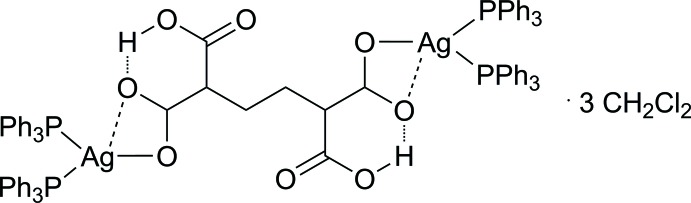


A further application of silver carboxyl­ate precursors includes their use for joining of bulk copper to produce metallic inter­connects, for example in microelectronic applications (Oestreicher *et al.*, 2012 ▸, 2013 ▸).

We anti­cipate that a metal oxide layer will need to be removed during such a silver-facilitated copper-joining process. Leaving some of the carboxyl groups of the silver precursor uncoordinated is expected to assist in this process. In the case of sparingly soluble silver carboxyl­ates, the solubility in common organic solvents can be increased through addition of phosphanes, such as triphenyl phosphane. In this context, the title compound (I) was prepared by the reaction of the disilver salt of butane-1,1,4,4-tetra­carboxyl acid (BTCA) with tri­phenyl­phosphane.

## Structural commentary   

**Figure 1:**
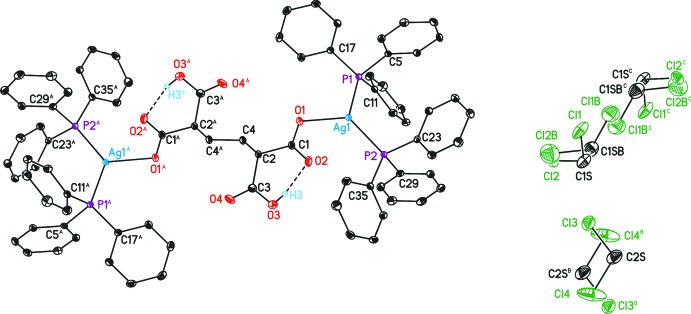
The mol­ecular structure of (I), with displacement ellipsoids drawn at the 30% probability level, including the intra­molecular hydrogen bonds. All non-O-bonded H atoms and the labels of the *o*-, *m*- and *p*-phenyl C atoms have been omitted for clarity. [Symmetry codes: (A) −*x*, −*y* + 1, −*z* + 2; (B) −*x* + 1, −*y* + 1, −*z*; (C) −*x* + 1, −*y*, −*z*.]

The title compound (I) crystallizes in the triclinic space group *P* 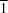. The asymmetric unit contains a half mol­ecule of butane-1,4-dicarb­oxy-1,4-di­carboxyl­ate bonded to a bis­(tri­phenyl­phosphan­yl)silver moiety and 1.5 mol­ecules of di­chloro­methane. The inversion centre to build up the whole disilver complex is located in the middle of the C4—C4′ bond (Fig. 1 ▸; (A): –x, –y + 1, –z + 2). The three mol­ecules of di­chloro­methane are also located on or nearby inversion centres (Fig. 1 ▸; C2*S*, (B): –x + 1, –y + 1, –z; C1*S*, C1*SB*, –x + 1, –y, –z; see *Refinement* section for details).

The anionic C_8_H_8_O_8_ moiety contains an intra­molecular hydrogen bond between the O3 atom of the HO_2_C-carb­oxy group and the O2 atom that is in inter­action with Ag1 (Fig. 1 ▸, Table 1 ▸), resulting in a boat-type conformation including the C1, C2 and C3 atoms, due to a synperiplanar torsion of the C1—O2 and C3—O3 bonds by 6.3 (2)°. Within the H-bearing carboxyl­ato substituent a distinction between the C,O single [1.321 (3) Å] and double [1.205 (3) Å] bonds can be observed. The C1 labeled carboxyl­ato group exhibits an unsymmetrically bidendate binding to Ag1. Therefore, O1 is, with 2.3305 (17) Å, σ-bonded, whereas O2 exhibits a weaker inter­action with an increased O2⋯Ag1 distance of 2.6872 (19) Å, probably due to the involvement in the hydrogen bond.

The Ag1 atom exhibits a somewhat distorted trigonal–planar P_2_O coordination environment, whereby the two phosphanes enclose an increased P—Ag—P angle of 128.56 (2)°, in contrast to the O1—Ag1—P angles of 117.69 (5) (P1) and 113.27 (5)° (P2). The weak inter­action to the O2 atom occurs below this AgP_2_ plane with an inter­action to the CO_2_ group of 67.38 (17)° with, however, two nearly equal C1—O1/O2 bond lengths of 1.251 (3) (O1) and 1.261 (3) Å (O2). Both phosphanes reveal an eclipsed conformation regarding the phenyl rings of 2.09 (10)°.

## Supra­molecular features   

**Figure 2:**
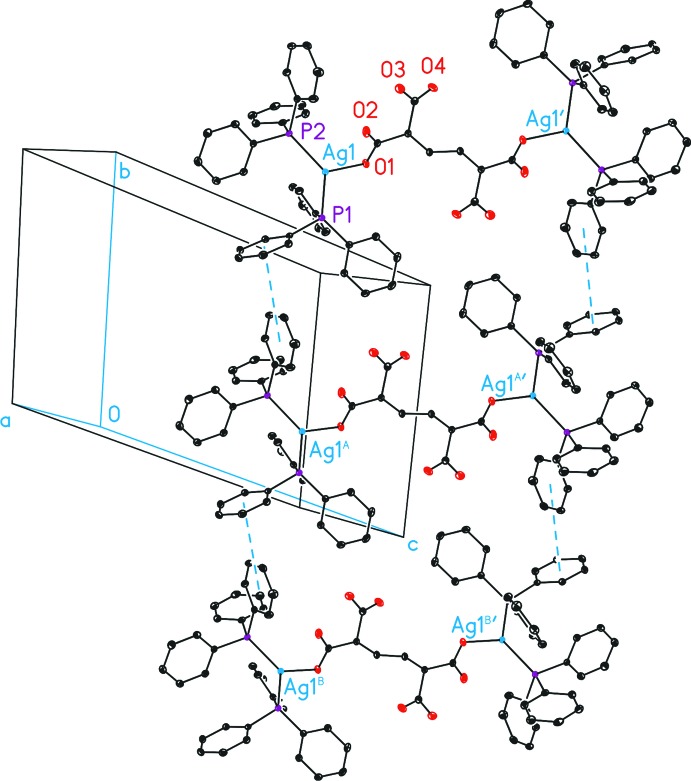
Inter­molecular *T*-shaped π–π inter­actions (blue) between the centroids (D) of the C5–C10 and C35–C40 labeled phenyl rings in the crystal structure of (I). All H atoms and solvent mol­ecules have been omitted for clarity. D—D = 4.8497 (16) Å; α = 77.40 (13) °. [Symmetry codes: (′) −*x*, −*y* + 1, −*z* + 2; (A) *x*, 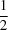 − *y*, *z* − 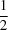; (B) *x*, *y*, *x* − 1.]

The packing of (I) consists of a layer-type structure parallel to (101), which is supported by weak *T*-shaped π–π inter­actions (Sinnokrot *et al.*, 2002 ▸) between the C5–C10 and the C35–C40 labeled phenyl rings with centroid–centroid distances of 4.8497 (16) Å [α = 77.40 (13)°] at both sides of the mol­ecules, forming a ladder-type arrangement parallel to [010] (Fig. 2 ▸). These ladders are packed along (101) through the phenyl rings, however, without showing any further π–π or C—H inter­actions.

One di­chloro­methane is stabilized by a non-classical hydrogen-bridge bond from C1*S*, as the hydrogen-bond donor, to the hydroxyl O3 atom (Table 1 ▸), which also acts as hydrogen-bridge bond donor in an intra­molecular classical bridge bond (see *Structural commentary*).

Further inter­molecular inter­actions involving hydrogen bonds or O⋯Ag inter­actions are not observed.

## Database survey   

In the CSD database (Groom & Allen, 2014 ▸; Version 5.36), only two acyclic silver tetra­carboxyl­ates with six-membered carbon backbones are reported. These are butane-1,2,3,4-tetra­carboxyl­ato silver compounds containing further nitro­gen and oxygen donor ligands, coordinating the silver ions either in a tetra­hedral or a *T*-shaped trigonal fashion (Sun *et al.*, 2010 ▸). Three aliphatic cyclo­hexane silver complexes with four to six carboxyl­ate groups are also known. Within those, the silver ions are also coordinated by additional ligands such as ammonia and water and possess distorted tetra­hedral coordination or *Y*-shaped coordination environments (Wang *et al.*, 2006 ▸, 2009 ▸). For six-membered unbranched acyclic silver di­carboxyl­ates derived from adipic acid, more crystal structures are reported compared to the respective tetra­carboxyl­ato derivatives. Several coordination geometries for the silver atoms are reported such as *T*-shaped (Wu *et al.*, 1995 ▸), tetra­hedral (Li *et al.*, 2011 ▸) or trigonal–planar environments (Liu *et al.*, 2009 ▸) containing nitro­gen, oxygen or sulfur donor ligands. To the best of our knowledge, the title compound (I) is the only example of a silver tetra­carboxyl­ate consisting of a six-membered carbon backbone and containing a silver–phospho­rus bond. In contrast to the title compound, which exists as a monomer presumably due to the steric shielding by triphenyl phosphane, all of the above-mentioned complexes exist as polymeric networks, formed by bridging through the different donor atoms of the ligands. For example, by using silver di­carboxyl­ates frequently the formation of dimeric sub-units can take place, which in turn results in the construction of polymeric systems (Wu *et al.*, 1995 ▸). Structures containing water mol­ecules coordinating to the Ag^I^ ions result in the formation of a further polymeric hydrogen bridge-bond network, also including carboxyl­ato moieties (Wang *et al.*, 2006 ▸, 2009 ▸).

## Synthesis and crystallization   

**Synthesis of butane-1,4-dicarboxyl-1,4-di­carboxyl­ato­di­silver(I):**

Potassium *tert-*butano­late (192 mg, 1.71 mmol) was added to a solution of butane-1,1,4,4-tetra­carboxyl acid (200 mg, 0.854 mmol) in 5 mL of tetra­hydro­furan. After stirring overnight at ambient temperature, the reaction mixture was filtered off and the residue was washed trice with tetra­hydro­furan (10 mL each) and dried under vacuum (yield 243 mg). Subsequently, the obtained colorless solid (243 mg, 0.783 mmol) was dissolved in water (15 mL) and added dropwise to a solution of silver nitrate (267 mg, 1.57 mmol) in water (8 mL). After 12 h of stirring the colorless precipitate was filtered off and washed twice with water (10 mL each) and dried in a desiccator. The desired colorless butane-1,4-dicarboxyl-1,4-di­carboxyl­atodisilver(I) was obtained in a yield of 71%, based on butane-1,1,4,4-tetra­carboxyl acid (271 mg, 0.608 mmol). Analysis calculated for C_8_H_8_Ag_2_O_8_ (447.88): C, 21.45; H, 1.80. Found: C, 21.49; H, 1.68. IR (KBr, cm^−1^): *ν* = 2977 (*m*), 2903 (*m*), 2461 (*m*), 1660 (*s*), 1549 (*vs*), 1380 (*s*), 1257 (*s*), 1069 (*s*), 955 (*m*), 711 (*m*).

**Synthesis of (μ-1,4-di­carb­oxy­butane-1,4-di­carboxyl­ato)bis[bis­(tri­phenyl­phosphane)silver(I)]:**

To a suspension of butane-1,4-dicarboxyl-1,4-di­carboxyl­atodisilver(I) (100 mg, 0.223 mmol) in 10 mL of tetra­hydro­furan, PPh_3_ (234 mg, 0.892 mmol) was added in a single portion at ambient temperature. After 12 h of stirring the reaction mixture was filtered through a pad of celite. After removal of all volatiles under reduced pressure, the title compound (I) was obtained as a colorless solid (275 mg, 0.184 mmol, 83% based on butane-1,4-dicarboxyl-1,4-di­carboxyl­atodisilver(I). Slow diffusion of pentane into a di­chloro­methane solution containing (I) at ambient temperature afforded colourless crystals of (I). M.p. 398 K (decomp.). ^1^H NMR (500 MHz, CDCl_3_, 298 K, p.p.m.): δ = 7.39–7.29 (*m*, 60H, C_6_H_5_), 2.93 (*m*, 2H, CH), 2.04 (*m*, 4H, CH_2_). ^13^C{^1^H} NMR (126 MHz, CDCl_3_, 298 K, p.p.m.): *δ* = 175.9 (*s*, C=O), 134.0 (*d*, ^2^ *J*_PC_ = 16.1 Hz, *o*-C_6_H_5_), 131.7 (*d*, ^1^ *J*_PC_ = 29.3 Hz, C^*i*^-C_6_H_5_), 130.6 (*s*, *p*-C_6_H_5_), 129.1 (*d*, ^3^ *J*_PC_ = 9.3 Hz, *m*-C_6_H_5_), 50.8 (*s*, CH), 29.6 (*s*, CH_2_). ^31^P{^1^H} NMR (203 MHz, CDCl_3_, 298 K, p.p.m.): *δ* = 10.3 (*s*). IR (KBr, cm^−1^): *ν* = 3108 (*w*), 2993 (*w*), 1751 (*s*), 1494 (*vs*), 1106 (*s*), 752 (*vs*), 701 (*vs*).

## Refinement   

Crystal data, data collection and structure refinement details are summarized in Table 2 ▸. C-bonded hydrogen atoms were placed in calculated positions and constrained to ride on their parent atoms with *U*_iso_(H) = 1.2*U*_eq_(C) and a C—H distance of 0.93 Å for aromatic (AFIX 43), 0.98 Å for methine (AFIX 13) and 0.97 Å for methyl­ene H atoms (AFIX 23). The same applies for the O-bonded H atom; however, the torsion angle was derived from electron density (AFIX 147). The structure contains three mol­ecules of di­chloro­methane as the solvent. Both crystallographically independent mol­ecules consist of two moieties each. One mol­ecule was refined as disordered over two positions (C1*S*; C1*SB*) with occupancies of 92.7 (2) and 7.3 (3)%, respectively. The less prevalent moiety of C1*SB* is located close to a crystallographic inversion centre and symmetry-related pairs are mutually exclusive. The second disordered mol­ecule is located directly atop of another inversion centre with an occupancy of 0.5 (Fig. 1 ▸), with the inversion centre located near the C2*S* and Cl1*B* atoms. The less-occupied methyl­ene chloride mol­ecule was restrained to have a geometry similar to that of its major moiety counterpart by using the SAME command. *U*_ij_ components of ADPs for C1*S* C1*SB* Cl1*B* and Cl2*B* were restrained to be similar if closer than 1.7 Å (SIMU restraint, McArdle, 1995 ▸; Sheldrick, 2008 ▸).

## Supplementary Material

Crystal structure: contains datablock(s) I, 1R. DOI: 10.1107/S2056989016000797/zl2653sup1.cif

Structure factors: contains datablock(s) I. DOI: 10.1107/S2056989016000797/zl2653Isup2.hkl

CCDC reference: 1447419

Additional supporting information:  crystallographic information; 3D view; checkCIF report

## Figures and Tables

**Table 1:** Hydrogen-bond geometry (Å, °)

*D*—H⋯*A*	*D*—H	H⋯*A*	*D*⋯*A*	*D*—H⋯*A*
O3—H3⋯O2	0.84	1.79	2.525 (3)	146
C1*S*—H1*S*1⋯O3^i^	0.99	2.53	2.997 (4)	108
C1*SB*—H1*S*4⋯O3^i^	0.99	2.46	3.04 (4)	117

Symmetry code: (i) 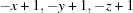.

**Table 2:** Experimental details

Crystal data	
Chemical formula	[Ag_2_(C_8_H_8_O_8_)(C_18_H_15_P)_4_]·3CH_2_Cl_2_
*M*_r_	1751.74
Crystal system, space group	Triclinic, *P* 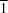
Temperature (K)	110
*a*, *b*, *c* (Å)	10.0279 (3), 12.9540 (4), 16.8190 (5)
α, β, γ (°)	112.306 (3), 96.080 (3), 103.601 (3)
*V* (Å^3^)	1917.80 (11)
*Z*	1
Radiation type	Mo *K*α
μ (mm^−1^)	0.86
Crystal size (mm)	0.3 × 0.3 × 0.2

Data collection	
Diffractometer	Oxford Gemini S
Absorption correction	Multi-scan (*CrysAlis RED*; Oxford Diffraction, 2006 ▸)
*T*_min_, *T*_max_	0.889, 1.000
No. of measured, independent and observed [*I* > 2σ(*I*)] reflections	21441, 8700, 7959
*R*_int_	0.024
(sin θ/λ)_max_ (Å^−1^)	0.680

Refinement	
*R*[*F* ^2^ > 2σ(*F* ^2^)], *wR*(*F* ^2^), *S*	0.034, 0.083, 1.06
No. of reflections	8700
No. of parameters	507
No. of restraints	27
H-atom treatment	H-atom parameters constrained
Δρ_max_, Δρ_min_ (e Å^−3^)	2.28, −0.61

Computer programs: *CrysAlis CCD* (Oxford Diffraction, 2006 ▸), *CrysAlis RED* (Oxford Diffraction, 2006 ▸), *SHELXS2013* (Sheldrick, 2008 ▸), *SHELXL2014* (Sheldrick, 2015 ▸), *ORTEP-3 for Windows* (Farrugia, 2012 ▸) and *SHELXTL* (Sheldrick, 2008 ▸), *WinGX* (Farrugia, 2012 ▸) and *publCIF* (Westrip, 2010 ▸).

## supplementary crystallographic information

### Crystal data

**Table:**

[Ag_2_(C_8_H_8_O_8_)(C_18_H_15_P)_4_]·3CH_2_Cl_2_	*Z* = 1
*M**_r_* = 1751.74	*F*(000) = 892
Triclinic, *P*1	*D*_x_ = 1.517 Mg m^−^^3^
*a* = 10.0279 (3) Å	Mo *K*α radiation, λ = 0.71073 Å
*b* = 12.9540 (4) Å	Cell parameters from 12088 reflections
*c* = 16.8190 (5) Å	θ = 3.4–28.8°
α = 112.306 (3)°	µ = 0.86 mm^−^^1^
β = 96.080 (3)°	*T* = 110 K
γ = 103.601 (3)°	Block, colorless
*V* = 1917.80 (11) Å^3^	0.3 × 0.3 × 0.2 mm

### Data collection

**Table:**

Oxford Gemini S diffractometer	*R*_int_ = 0.024
ω scans	θ_max_ = 28.9°, θ_min_ = 2.9°
Absorption correction: multi-scan (*CrysAlis RED*; Oxford Diffraction, 2006)	*h* = −13→12
*T*_min_ = 0.889, *T*_max_ = 1.000	*k* = −17→17
21441 measured reflections	*l* = −22→20
8700 independent reflections	2 standard reflections every 50 reflections
7959 reflections with *I* > 2σ(*I*)	intensity decay: none

### Refinement

**Table:**

Refinement on *F*^2^	Primary atom site location: structure-invariant direct methods
Least-squares matrix: full	Secondary atom site location: difference Fourier map
*R*[*F*^2^ > 2σ(*F*^2^)] = 0.034	Hydrogen site location: inferred from neighbouring sites
*wR*(*F*^2^) = 0.083	H-atom parameters constrained
*S* = 1.06	*w* = 1/[σ^2^(*F*_o_^2^) + (0.032*P*)^2^ + 2.6912*P*] where *P* = (*F*_o_^2^ + 2*F*_c_^2^)/3
8700 reflections	(Δ/σ)_max_ = 0.001
507 parameters	Δρ_max_ = 2.28 e Å^−^^3^
27 restraints	Δρ_min_ = −0.61 e Å^−^^3^

### Special details

**Table:**

Geometry. All e.s.d.'s (except the e.s.d. in the dihedral angle between two l.s. planes) are estimated using the full covariance matrix. The cell e.s.d.'s are taken into account individually in the estimation of e.s.d.'s in distances, angles and torsion angles; correlations between e.s.d.'s in cell parameters are only used when they are defined by crystal symmetry. An approximate (isotropic) treatment of cell e.s.d.'s is used for estimating e.s.d.'s involving l.s. planes.

### Fractional atomic coordinates and isotropic or equivalent isotropic displacement parameters (Å^2^)

**Table:**

	*x*	*y*	*z*	*U*_iso_*/*U*_eq_	Occ. (<1)
C1	0.1098 (3)	0.4419 (2)	0.82508 (15)	0.0190 (5)	
C2	0.0325 (2)	0.5106 (2)	0.88968 (15)	0.0174 (4)	
H2	−0.0699	0.4812	0.8615	0.021*	
C3	0.0822 (3)	0.6429 (2)	0.91901 (16)	0.0247 (5)	
C4	0.0521 (3)	0.4833 (2)	0.97147 (15)	0.0189 (5)	
H4A	0.0402	0.3987	0.9518	0.023*	
H4B	0.1489	0.5265	1.0070	0.023*	
C5	0.2398 (2)	−0.00432 (19)	0.61984 (14)	0.0158 (4)	
C6	0.1010 (3)	−0.0530 (2)	0.57113 (16)	0.0204 (5)	
H6	0.0276	−0.0297	0.5975	0.024*	
C7	0.0703 (3)	−0.1351 (2)	0.48448 (16)	0.0232 (5)	
H7	−0.0241	−0.1678	0.4518	0.028*	
C8	0.1774 (3)	−0.1698 (2)	0.44511 (16)	0.0243 (5)	
H8	0.1562	−0.2258	0.3857	0.029*	
C9	0.3145 (3)	−0.1225 (2)	0.49301 (16)	0.0231 (5)	
H9	0.3875	−0.1461	0.4663	0.028*	
C10	0.3465 (3)	−0.0403 (2)	0.58043 (16)	0.0193 (5)	
H10	0.4409	−0.0088	0.6131	0.023*	
C11	0.4578 (2)	0.1469 (2)	0.77394 (15)	0.0173 (4)	
C12	0.5538 (3)	0.2188 (2)	0.74783 (17)	0.0230 (5)	
H12	0.5203	0.2509	0.7103	0.028*	
C13	0.6972 (3)	0.2435 (2)	0.77632 (17)	0.0256 (5)	
H13	0.7616	0.2911	0.7575	0.031*	
C14	0.7467 (3)	0.1989 (2)	0.83205 (18)	0.0280 (6)	
H14	0.8450	0.2153	0.8512	0.034*	
C15	0.6527 (3)	0.1299 (3)	0.86010 (19)	0.0314 (6)	
H15	0.6869	0.1010	0.8997	0.038*	
C16	0.5089 (3)	0.1029 (2)	0.83059 (17)	0.0251 (5)	
H16	0.4451	0.0544	0.8491	0.030*	
C17	0.1720 (2)	0.0459 (2)	0.78922 (15)	0.0172 (4)	
C18	0.1697 (3)	−0.0662 (2)	0.78067 (16)	0.0222 (5)	
H18	0.2230	−0.1071	0.7440	0.027*	
C19	0.0898 (3)	−0.1177 (2)	0.82571 (17)	0.0269 (5)	
H19	0.0880	−0.1940	0.8196	0.032*	
C20	0.0124 (3)	−0.0581 (2)	0.87957 (17)	0.0266 (5)	
H20	−0.0423	−0.0938	0.9103	0.032*	
C21	0.0144 (3)	0.0534 (2)	0.88891 (17)	0.0256 (5)	
H21	−0.0382	0.0942	0.9263	0.031*	
C22	0.0939 (3)	0.1055 (2)	0.84336 (16)	0.0215 (5)	
H22	0.0948	0.1816	0.8493	0.026*	
C23	0.2912 (2)	0.2770 (2)	0.50087 (15)	0.0162 (4)	
C24	0.2945 (3)	0.1622 (2)	0.47850 (16)	0.0207 (5)	
H24	0.3061	0.1354	0.5234	0.025*	
C25	0.2812 (3)	0.0869 (2)	0.39126 (17)	0.0239 (5)	
H25	0.2846	0.0094	0.3768	0.029*	
C26	0.2629 (3)	0.1250 (2)	0.32544 (17)	0.0259 (5)	
H26	0.2542	0.0739	0.2658	0.031*	
C27	0.2574 (3)	0.2385 (2)	0.34706 (17)	0.0276 (6)	
H27	0.2436	0.2644	0.3019	0.033*	
C28	0.2719 (3)	0.3141 (2)	0.43393 (16)	0.0220 (5)	
H28	0.2686	0.3917	0.4480	0.026*	
C29	0.5028 (2)	0.4324 (2)	0.65811 (15)	0.0175 (4)	
C30	0.5974 (3)	0.4021 (2)	0.60549 (16)	0.0212 (5)	
H30	0.5636	0.3484	0.5454	0.025*	
C31	0.7417 (3)	0.4499 (2)	0.64032 (18)	0.0245 (5)	
H31	0.8060	0.4283	0.6041	0.029*	
C32	0.7910 (3)	0.5285 (2)	0.72730 (18)	0.0269 (5)	
H32	0.8893	0.5623	0.7507	0.032*	
C33	0.6974 (3)	0.5585 (2)	0.78088 (17)	0.0285 (6)	
H33	0.7319	0.6123	0.8409	0.034*	
C34	0.5535 (3)	0.5101 (2)	0.74688 (16)	0.0240 (5)	
H34	0.4896	0.5297	0.7838	0.029*	
C35	0.2611 (2)	0.4971 (2)	0.61527 (14)	0.0171 (4)	
C36	0.1241 (3)	0.4982 (2)	0.61812 (18)	0.0252 (5)	
H36	0.0602	0.4370	0.6253	0.030*	
C37	0.0796 (3)	0.5889 (2)	0.6105 (2)	0.0303 (6)	
H37	−0.0151	0.5885	0.6114	0.036*	
C38	0.1724 (3)	0.6791 (2)	0.60168 (16)	0.0253 (5)	
H38	0.1413	0.7401	0.5955	0.030*	
C39	0.3101 (3)	0.6805 (2)	0.60184 (17)	0.0248 (5)	
H39	0.3749	0.7438	0.5977	0.030*	
C40	0.3546 (3)	0.5896 (2)	0.60809 (17)	0.0223 (5)	
H40	0.4495	0.5906	0.6075	0.027*	
P1	0.27220 (6)	0.11490 (5)	0.72910 (4)	0.01518 (12)	
P2	0.31285 (6)	0.37213 (5)	0.61693 (4)	0.01519 (12)	
Ag1	0.22109 (2)	0.27730 (2)	0.70903 (2)	0.01622 (6)	
O1	0.04100 (19)	0.34138 (15)	0.76927 (11)	0.0239 (4)	
O2	0.23936 (19)	0.48772 (17)	0.83418 (12)	0.0301 (4)	
O3	0.2146 (2)	0.68777 (18)	0.91847 (15)	0.0409 (5)	
H3	0.2542	0.6350	0.9052	0.061*	
O4	0.0070 (2)	0.70357 (16)	0.94320 (13)	0.0333 (4)	
C1S	0.4951 (4)	0.1726 (4)	0.0721 (3)	0.0525 (11)	0.928 (2)
H1S1	0.5689	0.1417	0.0457	0.063*	0.928 (2)
H1S2	0.4983	0.2447	0.0638	0.063*	0.928 (2)
Cl1	0.33022 (9)	0.06889 (12)	0.01685 (7)	0.0647 (4)	0.928 (2)
Cl2	0.53101 (12)	0.20717 (8)	0.18454 (6)	0.0474 (3)	0.928 (2)
C1SB	0.525 (5)	0.125 (3)	0.069 (3)	0.069 (7)	0.072 (2)
H1S3	0.6161	0.1301	0.1016	0.083*	0.072 (2)
H1S4	0.5460	0.1770	0.0381	0.083*	0.072 (2)
Cl1B	0.451 (2)	−0.0184 (18)	−0.0110 (13)	0.088 (6)	0.072 (2)
Cl2B	0.429 (3)	0.183 (2)	0.1455 (16)	0.112 (7)	0.072 (2)
C2S	0.5061 (9)	0.4570 (8)	−0.0394 (6)	0.060 (2)	0.5
H2S1	0.4788	0.4427	−0.1019	0.072*	0.5
H2S2	0.5940	0.4361	−0.0319	0.072*	0.5
Cl3	0.3762 (2)	0.3690 (2)	−0.01470 (16)	0.0548 (5)	0.5
Cl4	0.5373 (5)	0.6054 (2)	0.0271 (3)	0.1237 (17)	0.5

### Atomic displacement parameters (Å^2^)

**Table:**

	*U*^11^	*U*^22^	*U*^33^	*U*^12^	*U*^13^	*U*^23^
C1	0.0254 (12)	0.0211 (12)	0.0169 (11)	0.0116 (9)	0.0102 (9)	0.0105 (9)
C2	0.0230 (11)	0.0167 (11)	0.0149 (10)	0.0077 (9)	0.0082 (9)	0.0070 (9)
C3	0.0380 (14)	0.0213 (13)	0.0158 (11)	0.0076 (11)	0.0087 (10)	0.0087 (10)
C4	0.0236 (11)	0.0206 (12)	0.0174 (11)	0.0112 (9)	0.0094 (9)	0.0092 (9)
C5	0.0237 (11)	0.0118 (10)	0.0156 (10)	0.0066 (9)	0.0074 (9)	0.0080 (8)
C6	0.0230 (12)	0.0206 (12)	0.0214 (11)	0.0085 (9)	0.0081 (9)	0.0109 (10)
C7	0.0250 (12)	0.0215 (12)	0.0210 (12)	0.0037 (10)	0.0011 (9)	0.0097 (10)
C8	0.0388 (14)	0.0169 (12)	0.0179 (11)	0.0099 (10)	0.0077 (10)	0.0067 (9)
C9	0.0336 (13)	0.0204 (12)	0.0227 (12)	0.0149 (10)	0.0149 (10)	0.0105 (10)
C10	0.0239 (12)	0.0166 (11)	0.0216 (11)	0.0089 (9)	0.0086 (9)	0.0098 (9)
C11	0.0211 (11)	0.0149 (11)	0.0165 (10)	0.0072 (9)	0.0057 (9)	0.0056 (9)
C12	0.0258 (12)	0.0201 (12)	0.0247 (12)	0.0061 (10)	0.0051 (10)	0.0117 (10)
C13	0.0241 (12)	0.0200 (12)	0.0304 (13)	0.0032 (10)	0.0086 (10)	0.0092 (10)
C14	0.0210 (12)	0.0267 (14)	0.0312 (14)	0.0076 (10)	0.0020 (10)	0.0074 (11)
C15	0.0312 (14)	0.0380 (16)	0.0314 (14)	0.0139 (12)	0.0034 (11)	0.0200 (13)
C16	0.0264 (13)	0.0275 (13)	0.0270 (13)	0.0088 (10)	0.0069 (10)	0.0164 (11)
C17	0.0218 (11)	0.0156 (11)	0.0163 (10)	0.0055 (9)	0.0061 (9)	0.0084 (9)
C18	0.0327 (13)	0.0187 (12)	0.0195 (11)	0.0104 (10)	0.0103 (10)	0.0096 (9)
C19	0.0390 (15)	0.0187 (12)	0.0241 (12)	0.0046 (11)	0.0091 (11)	0.0120 (10)
C20	0.0293 (13)	0.0307 (14)	0.0224 (12)	0.0039 (11)	0.0087 (10)	0.0160 (11)
C21	0.0269 (13)	0.0335 (14)	0.0223 (12)	0.0117 (11)	0.0117 (10)	0.0148 (11)
C22	0.0258 (12)	0.0214 (12)	0.0227 (12)	0.0116 (10)	0.0094 (10)	0.0110 (10)
C23	0.0171 (10)	0.0168 (11)	0.0170 (10)	0.0056 (8)	0.0077 (8)	0.0080 (9)
C24	0.0273 (12)	0.0197 (12)	0.0211 (11)	0.0100 (10)	0.0094 (9)	0.0120 (10)
C25	0.0299 (13)	0.0182 (12)	0.0257 (12)	0.0095 (10)	0.0107 (10)	0.0087 (10)
C26	0.0306 (13)	0.0236 (13)	0.0195 (12)	0.0069 (10)	0.0071 (10)	0.0052 (10)
C27	0.0398 (15)	0.0277 (14)	0.0190 (12)	0.0108 (11)	0.0060 (11)	0.0134 (10)
C28	0.0286 (13)	0.0197 (12)	0.0216 (12)	0.0094 (10)	0.0064 (10)	0.0112 (10)
C29	0.0198 (11)	0.0173 (11)	0.0217 (11)	0.0075 (9)	0.0062 (9)	0.0131 (9)
C30	0.0235 (12)	0.0199 (12)	0.0240 (12)	0.0098 (10)	0.0081 (10)	0.0106 (10)
C31	0.0212 (12)	0.0256 (13)	0.0338 (14)	0.0100 (10)	0.0094 (10)	0.0173 (11)
C32	0.0223 (12)	0.0292 (14)	0.0342 (14)	0.0052 (10)	0.0022 (10)	0.0209 (12)
C33	0.0331 (14)	0.0281 (14)	0.0207 (12)	0.0033 (11)	0.0002 (10)	0.0116 (11)
C34	0.0286 (13)	0.0253 (13)	0.0215 (12)	0.0087 (10)	0.0088 (10)	0.0121 (10)
C35	0.0247 (12)	0.0156 (11)	0.0150 (10)	0.0091 (9)	0.0077 (9)	0.0080 (9)
C36	0.0249 (12)	0.0216 (13)	0.0360 (14)	0.0094 (10)	0.0107 (11)	0.0167 (11)
C37	0.0270 (13)	0.0295 (14)	0.0432 (16)	0.0162 (11)	0.0109 (12)	0.0190 (12)
C38	0.0389 (14)	0.0198 (12)	0.0222 (12)	0.0156 (11)	0.0084 (11)	0.0096 (10)
C39	0.0366 (14)	0.0165 (12)	0.0266 (13)	0.0087 (10)	0.0117 (11)	0.0127 (10)
C40	0.0264 (12)	0.0196 (12)	0.0271 (12)	0.0095 (10)	0.0121 (10)	0.0132 (10)
P1	0.0194 (3)	0.0141 (3)	0.0162 (3)	0.0073 (2)	0.0073 (2)	0.0084 (2)
P2	0.0195 (3)	0.0148 (3)	0.0162 (3)	0.0079 (2)	0.0082 (2)	0.0089 (2)
Ag1	0.02215 (9)	0.01473 (9)	0.01672 (9)	0.00874 (7)	0.00930 (6)	0.00856 (7)
O1	0.0280 (9)	0.0196 (9)	0.0237 (9)	0.0090 (7)	0.0139 (7)	0.0053 (7)
O2	0.0226 (9)	0.0352 (11)	0.0260 (9)	0.0061 (8)	0.0114 (7)	0.0060 (8)
O3	0.0440 (12)	0.0228 (10)	0.0449 (12)	−0.0002 (9)	0.0212 (10)	0.0058 (9)
O4	0.0535 (13)	0.0207 (10)	0.0303 (10)	0.0192 (9)	0.0126 (9)	0.0100 (8)
C1S	0.040 (2)	0.069 (3)	0.046 (2)	−0.0016 (19)	0.0037 (16)	0.034 (2)
Cl1	0.0314 (5)	0.1057 (9)	0.0486 (6)	−0.0137 (5)	−0.0060 (4)	0.0476 (6)
Cl2	0.0634 (6)	0.0420 (5)	0.0313 (4)	0.0193 (4)	0.0035 (4)	0.0091 (4)
C1SB	0.063 (9)	0.077 (9)	0.062 (9)	0.007 (9)	0.008 (8)	0.033 (9)
Cl1B	0.124 (15)	0.080 (11)	0.075 (10)	0.032 (12)	0.019 (11)	0.047 (9)
Cl2B	0.101 (11)	0.108 (11)	0.106 (11)	0.021 (10)	0.014 (10)	0.029 (10)
C2S	0.044 (4)	0.063 (6)	0.082 (6)	0.011 (4)	0.023 (4)	0.041 (5)
Cl3	0.0507 (11)	0.0616 (14)	0.0619 (12)	0.0195 (10)	0.0221 (10)	0.0321 (11)
Cl4	0.165 (4)	0.0433 (15)	0.109 (3)	−0.009 (2)	−0.068 (3)	0.0238 (16)

### Geometric parameters (Å, º)

**Table:**

C1—O1	1.251 (3)	C24—C25	1.390 (3)
C1—O2	1.261 (3)	C24—H24	0.9500
C1—C2	1.531 (3)	C25—C26	1.385 (4)
C2—C3	1.527 (3)	C25—H25	0.9500
C2—C4	1.550 (3)	C26—C27	1.390 (4)
C2—H2	1.0000	C26—H26	0.9500
C3—O4	1.205 (3)	C27—C28	1.385 (4)
C3—O3	1.321 (3)	C27—H27	0.9500
C4—C4^i^	1.520 (4)	C28—H28	0.9500
C4—H4A	0.9900	C29—C30	1.388 (3)
C4—H4B	0.9900	C29—C34	1.399 (3)
C5—C10	1.397 (3)	C29—P2	1.825 (2)
C5—C6	1.400 (3)	C30—C31	1.395 (3)
C5—P1	1.831 (2)	C30—H30	0.9500
C6—C7	1.387 (3)	C31—C32	1.379 (4)
C6—H6	0.9500	C31—H31	0.9500
C7—C8	1.395 (4)	C32—C33	1.389 (4)
C7—H7	0.9500	C32—H32	0.9500
C8—C9	1.382 (4)	C33—C34	1.389 (4)
C8—H8	0.9500	C33—H33	0.9500
C9—C10	1.396 (3)	C34—H34	0.9500
C9—H9	0.9500	C35—C36	1.384 (3)
C10—H10	0.9500	C35—C40	1.391 (3)
C11—C16	1.393 (3)	C35—P2	1.821 (2)
C11—C12	1.402 (3)	C36—C37	1.394 (4)
C11—P1	1.819 (2)	C36—H36	0.9500
C12—C13	1.386 (4)	C37—C38	1.380 (4)
C12—H12	0.9500	C37—H37	0.9500
C13—C14	1.381 (4)	C38—C39	1.376 (4)
C13—H13	0.9500	C38—H38	0.9500
C14—C15	1.389 (4)	C39—C40	1.389 (3)
C14—H14	0.9500	C39—H39	0.9500
C15—C16	1.389 (4)	C40—H40	0.9500
C15—H15	0.9500	P1—Ag1	2.4109 (6)
C16—H16	0.9500	P2—Ag1	2.4433 (6)
C17—C22	1.393 (3)	Ag1—O1	2.3305 (17)
C17—C18	1.398 (3)	O3—H3	0.8400
C17—P1	1.819 (2)	C1S—Cl2	1.744 (4)
C18—C19	1.387 (3)	C1S—Cl1	1.756 (4)
C18—H18	0.9500	C1S—H1S1	0.9900
C19—C20	1.385 (4)	C1S—H1S2	0.9900
C19—H19	0.9500	C1SB—Cl2B	1.73 (2)
C20—C21	1.387 (4)	C1SB—Cl1B	1.75 (2)
C20—H20	0.9500	C1SB—H1S3	0.9900
C21—C22	1.395 (3)	C1SB—H1S4	0.9900
C21—H21	0.9500	C2S—Cl3	1.716 (8)
C22—H22	0.9500	C2S—Cl4	1.748 (10)
C23—C28	1.395 (3)	C2S—H2S1	0.9900
C23—C24	1.398 (3)	C2S—H2S2	0.9900
C23—P2	1.826 (2)		
			
O1—C1—O2	124.4 (2)	C25—C26—C27	119.7 (2)
O1—C1—C2	117.4 (2)	C25—C26—H26	120.2
O2—C1—C2	118.1 (2)	C27—C26—H26	120.2
C3—C2—C1	115.15 (19)	C28—C27—C26	120.5 (2)
C3—C2—C4	109.22 (19)	C28—C27—H27	119.7
C1—C2—C4	106.96 (18)	C26—C27—H27	119.7
C3—C2—H2	108.4	C27—C28—C23	120.3 (2)
C1—C2—H2	108.4	C27—C28—H28	119.8
C4—C2—H2	108.4	C23—C28—H28	119.8
O4—C3—O3	121.6 (2)	C30—C29—C34	119.4 (2)
O4—C3—C2	122.5 (2)	C30—C29—P2	122.57 (19)
O3—C3—C2	115.8 (2)	C34—C29—P2	118.00 (18)
C4^i^—C4—C2	112.2 (2)	C29—C30—C31	120.4 (2)
C4^i^—C4—H4A	109.2	C29—C30—H30	119.8
C2—C4—H4A	109.2	C31—C30—H30	119.8
C4^i^—C4—H4B	109.2	C32—C31—C30	119.9 (2)
C2—C4—H4B	109.2	C32—C31—H31	120.0
H4A—C4—H4B	107.9	C30—C31—H31	120.0
C10—C5—C6	119.2 (2)	C31—C32—C33	120.2 (2)
C10—C5—P1	123.58 (18)	C31—C32—H32	119.9
C6—C5—P1	116.88 (17)	C33—C32—H32	119.9
C7—C6—C5	120.2 (2)	C32—C33—C34	120.1 (2)
C7—C6—H6	119.9	C32—C33—H33	119.9
C5—C6—H6	119.9	C34—C33—H33	119.9
C6—C7—C8	120.3 (2)	C33—C34—C29	119.9 (2)
C6—C7—H7	119.8	C33—C34—H34	120.0
C8—C7—H7	119.8	C29—C34—H34	120.0
C9—C8—C7	119.7 (2)	C36—C35—C40	119.1 (2)
C9—C8—H8	120.2	C36—C35—P2	119.20 (18)
C7—C8—H8	120.2	C40—C35—P2	121.65 (18)
C8—C9—C10	120.5 (2)	C35—C36—C37	120.1 (2)
C8—C9—H9	119.8	C35—C36—H36	119.9
C10—C9—H9	119.8	C37—C36—H36	119.9
C9—C10—C5	120.0 (2)	C38—C37—C36	120.3 (2)
C9—C10—H10	120.0	C38—C37—H37	119.8
C5—C10—H10	120.0	C36—C37—H37	119.8
C16—C11—C12	118.9 (2)	C39—C38—C37	119.8 (2)
C16—C11—P1	124.05 (18)	C39—C38—H38	120.1
C12—C11—P1	117.01 (18)	C37—C38—H38	120.1
C13—C12—C11	120.5 (2)	C38—C39—C40	120.2 (2)
C13—C12—H12	119.8	C38—C39—H39	119.9
C11—C12—H12	119.8	C40—C39—H39	119.9
C14—C13—C12	120.1 (2)	C39—C40—C35	120.4 (2)
C14—C13—H13	119.9	C39—C40—H40	119.8
C12—C13—H13	119.9	C35—C40—H40	119.8
C13—C14—C15	119.9 (2)	C11—P1—C17	107.82 (11)
C13—C14—H14	120.0	C11—P1—C5	103.98 (11)
C15—C14—H14	120.0	C17—P1—C5	103.48 (10)
C14—C15—C16	120.4 (2)	C11—P1—Ag1	112.08 (8)
C14—C15—H15	119.8	C17—P1—Ag1	120.40 (8)
C16—C15—H15	119.8	C5—P1—Ag1	107.53 (7)
C15—C16—C11	120.2 (2)	C35—P2—C29	102.98 (11)
C15—C16—H16	119.9	C35—P2—C23	104.23 (10)
C11—C16—H16	119.9	C29—P2—C23	104.33 (11)
C22—C17—C18	119.6 (2)	C35—P2—Ag1	120.17 (7)
C22—C17—P1	119.02 (17)	C29—P2—Ag1	106.98 (7)
C18—C17—P1	121.32 (18)	C23—P2—Ag1	116.33 (7)
C19—C18—C17	120.1 (2)	O1—Ag1—P1	117.69 (5)
C19—C18—H18	120.0	O1—Ag1—P2	113.27 (5)
C17—C18—H18	120.0	P1—Ag1—P2	128.56 (2)
C20—C19—C18	120.1 (2)	C1—O1—Ag1	100.12 (14)
C20—C19—H19	120.0	C3—O3—H3	109.5
C18—C19—H19	120.0	Cl2—C1S—Cl1	112.6 (2)
C19—C20—C21	120.3 (2)	Cl2—C1S—H1S1	109.1
C19—C20—H20	119.8	Cl1—C1S—H1S1	109.1
C21—C20—H20	119.8	Cl2—C1S—H1S2	109.1
C20—C21—C22	119.9 (2)	Cl1—C1S—H1S2	109.1
C20—C21—H21	120.0	H1S1—C1S—H1S2	107.8
C22—C21—H21	120.0	Cl2B—C1SB—Cl1B	118 (2)
C17—C22—C21	119.9 (2)	Cl2B—C1SB—H1S3	107.8
C17—C22—H22	120.0	Cl1B—C1SB—H1S3	107.8
C21—C22—H22	120.0	Cl2B—C1SB—H1S4	107.8
C28—C23—C24	118.8 (2)	Cl1B—C1SB—H1S4	107.8
C28—C23—P2	122.78 (18)	H1S3—C1SB—H1S4	107.1
C24—C23—P2	118.41 (17)	Cl3—C2S—Cl4	112.3 (5)
C25—C24—C23	120.7 (2)	Cl3—C2S—H2S1	109.1
C25—C24—H24	119.7	Cl4—C2S—H2S1	109.1
C23—C24—H24	119.7	Cl3—C2S—H2S2	109.1
C26—C25—C24	120.0 (2)	Cl4—C2S—H2S2	109.1
C26—C25—H25	120.0	H2S1—C2S—H2S2	107.9
C24—C25—H25	120.0		
			
O1—C1—C2—C3	−147.4 (2)	C30—C29—C34—C33	1.8 (4)
O2—C1—C2—C3	35.8 (3)	P2—C29—C34—C33	−179.70 (19)
O1—C1—C2—C4	91.0 (2)	C40—C35—C36—C37	2.4 (4)
O2—C1—C2—C4	−85.7 (3)	P2—C35—C36—C37	−175.4 (2)
C1—C2—C3—O4	153.9 (2)	C35—C36—C37—C38	−1.2 (4)
C4—C2—C3—O4	−85.8 (3)	C36—C37—C38—C39	−1.1 (4)
C1—C2—C3—O3	−27.7 (3)	C37—C38—C39—C40	2.0 (4)
C4—C2—C3—O3	92.6 (2)	C38—C39—C40—C35	−0.8 (4)
C3—C2—C4—C4^i^	71.3 (3)	C36—C35—C40—C39	−1.5 (4)
C1—C2—C4—C4^i^	−163.5 (2)	P2—C35—C40—C39	176.25 (19)
C10—C5—C6—C7	−0.6 (3)	C16—C11—P1—C17	12.2 (2)
P1—C5—C6—C7	173.30 (18)	C12—C11—P1—C17	−168.95 (18)
C5—C6—C7—C8	0.0 (4)	C16—C11—P1—C5	−97.2 (2)
C6—C7—C8—C9	0.3 (4)	C12—C11—P1—C5	81.6 (2)
C7—C8—C9—C10	0.0 (4)	C16—C11—P1—Ag1	146.97 (19)
C8—C9—C10—C5	−0.7 (3)	C12—C11—P1—Ag1	−34.2 (2)
C6—C5—C10—C9	1.0 (3)	C22—C17—P1—C11	112.8 (2)
P1—C5—C10—C9	−172.51 (18)	C18—C17—P1—C11	−68.5 (2)
C16—C11—C12—C13	1.5 (4)	C22—C17—P1—C5	−137.45 (19)
P1—C11—C12—C13	−177.4 (2)	C18—C17—P1—C5	41.2 (2)
C11—C12—C13—C14	−1.1 (4)	C22—C17—P1—Ag1	−17.5 (2)
C12—C13—C14—C15	−0.5 (4)	C18—C17—P1—Ag1	161.23 (17)
C13—C14—C15—C16	1.7 (4)	C10—C5—P1—C11	−10.3 (2)
C14—C15—C16—C11	−1.3 (4)	C6—C5—P1—C11	176.06 (17)
C12—C11—C16—C15	−0.3 (4)	C10—C5—P1—C17	−122.87 (19)
P1—C11—C16—C15	178.5 (2)	C6—C5—P1—C17	63.48 (19)
C22—C17—C18—C19	0.2 (4)	C10—C5—P1—Ag1	108.71 (18)
P1—C17—C18—C19	−178.5 (2)	C6—C5—P1—Ag1	−64.94 (18)
C17—C18—C19—C20	−0.3 (4)	C36—C35—P2—C29	−157.1 (2)
C18—C19—C20—C21	−0.1 (4)	C40—C35—P2—C29	25.2 (2)
C19—C20—C21—C22	0.5 (4)	C36—C35—P2—C23	94.2 (2)
C18—C17—C22—C21	0.2 (4)	C40—C35—P2—C23	−83.5 (2)
P1—C17—C22—C21	178.93 (19)	C36—C35—P2—Ag1	−38.4 (2)
C20—C21—C22—C17	−0.5 (4)	C40—C35—P2—Ag1	143.93 (17)
C28—C23—C24—C25	1.0 (4)	C30—C29—P2—C35	−110.1 (2)
P2—C23—C24—C25	−178.78 (19)	C34—C29—P2—C35	71.4 (2)
C23—C24—C25—C26	−0.6 (4)	C30—C29—P2—C23	−1.5 (2)
C24—C25—C26—C27	−0.3 (4)	C34—C29—P2—C23	−179.95 (18)
C25—C26—C27—C28	0.9 (4)	C30—C29—P2—Ag1	122.33 (18)
C26—C27—C28—C23	−0.5 (4)	C34—C29—P2—Ag1	−56.13 (19)
C24—C23—C28—C27	−0.5 (4)	C28—C23—P2—C35	13.2 (2)
P2—C23—C28—C27	179.3 (2)	C24—C23—P2—C35	−167.02 (19)
C34—C29—C30—C31	−1.0 (3)	C28—C23—P2—C29	−94.5 (2)
P2—C29—C30—C31	−179.43 (18)	C24—C23—P2—C29	85.3 (2)
C29—C30—C31—C32	−0.5 (4)	C28—C23—P2—Ag1	147.93 (18)
C30—C31—C32—C33	1.3 (4)	C24—C23—P2—Ag1	−32.3 (2)
C31—C32—C33—C34	−0.5 (4)	O2—C1—O1—Ag1	4.6 (3)
C32—C33—C34—C29	−1.1 (4)	C2—C1—O1—Ag1	−171.92 (16)

Symmetry code: (i) −*x*, −*y*+1, −*z*+2.

### Hydrogen-bond geometry (Å, º)

**Table:**

*D*—H···*A*	*D*—H	H···*A*	*D*···*A*	*D*—H···*A*
O3—H3···O2	0.84	1.79	2.525 (3)	146
C1*S*—H1*S*1···O3^ii^	0.99	2.53	2.997 (4)	108
C1*SB*—H1*S*4···O3^ii^	0.99	2.46	3.04 (4)	117

Symmetry code: (ii) −*x*+1, −*y*+1, −*z*+1.
